# Chronic inflammation and apoptosis propagate in ischemic cerebellum and heart of non-human primates

**DOI:** 10.18632/oncotarget.18330

**Published:** 2017-06-01

**Authors:** Sandra A. Acosta, Sherwin Mashkouri, Diana Nwokoye, Jea Y. Lee, Cesar V. Borlongan

**Affiliations:** ^1^ Department of Neurosurgery and Brain Repair, University of South Florida Morsani College of Medicine, Tampa, Florida, USA

**Keywords:** secondary injury, neurodegeneration, cell death, Purkinje cells, Pathology Section

## Abstract

The major pathological consequences of cerebral ischemia are characterized by neurological deficits commonly ascribed to the infarcted tissue and its surrounding region, however, brain areas, as well as peripheral organs, distal from the original injury may manifest as subtle disease sequelae that can increase the risks of co-morbidities complicating the disease symptoms. To evaluate the vulnerability of the cerebellum and the heart to secondary injuries in the late stage of transient global ischemia (TGI) model in non-human primates (NHP), brain and heart tissues were collected at six months post-TGI. Unbiased stereological analyses of immunostained tissues showed significant Purkinje cells loss in lobule III and lobule IX of the TGI cerebellum relative to sham cerebellum, with corresponding upregulation of inflammatory and apoptotic cells. Similarly, TGI hearts revealed significant activation of inflammatory and apoptotic cells relative to sham hearts. Aberrant inflammation and apoptosis in the cerebellum and the heart of chronic TGI-exposed NHPs suggest distal secondary injuries manifesting both centrally and peripherally. These results advance our understanding on the sustained propagation of chronic secondary injuries after TGI, highlighting the need to develop therapeutic interventions targeting the brain, as well as the heart, in order to abrogate cerebral ischemia and its related co-morbidities.

## INTRODUCTION

Global ischemic injury caused by circulatory arrest has long been associated with physical changes in the human brain [1]. To date, these brain tissue modifications that follow global ischemia have been studied using both nonsurgical [2, 3] and surgical models [4]. Recently, minimally invasive surgical procedures have allowed researchers to safely and efficiently produce transient global ischemic injury in animal models [5]. Multiple studies have demonstrated that global ischemia is directly associated with localized neuronal damage in vulnerable brain areas such as the hippocampus and neocortex [5, 6].

A pathologic link may exist between neuronal death caused by cerebral ischemia and subsequent cardiac myocyte vulnerability [7-9]. Indeed, cellular death signals in cardiac tissue are upregulated following cerebral ischemia in both *in vitro* experiments and *in vivo* rodent models [7, 10]. These observations potentially explain the notable percentage of cardiac-related deaths in humans within 3 months of ischemic cerebral injury [7, 11, 12]. Recently, we provided a proof-of-concept that cerebral ischemia produces secondary cell damage in distal organs, especially the heart [7], using the ischemic stroke model in rodents. The mechanism of action was explored by exposing primary rat neuronal cells to oxygen-glucose deprivation (OGD) that caused a significant decrease in cellular vitality and mitochondrial activity in rat cardiomyocytes [7]. Our *in vivo* results further demonstrated that ischemic stroke models induced rat cardiomyocyte cell death [7]. Altogether, these results revealed that all cell death markers were detectable in both the brain and the heart after ischemic stroke, advancing the concept of a pathological link between the cerebrovascular and cardiovascular diseases.

Cerebrovascular and cardiovascular disease share several predisposing factors including hypertension, diabetes, hyperlipidemia, and family history of heart disease [13-21]. Research has shown a higher likelihood of cardiac cell death following an ischemic cerebrovascular episode, which is thought to be due to elevated levels of plasma catecholamines and cardiac enzymes, such as troponin and creatine phosphokinase [11, 12]. In addition, extreme ischemic events have been linked to increased levels of brain natriuretic peptide (BNP) [13-21]. The increased presence of this biomarker following ischemia suggests a crosstalk between the brain and heart after cerebral ischemia [19-22]. Furthermore, inflammation following ischemic stroke may serve as another pathological pathway connecting the brain and the heart. In particular, C-reactive protein (CRP) which is elevated during periods of inflammation within the body, presents as the specific factor found to be associated with the risk of new cardiovascular events in stroke patients [12], implicating inflammation as a key factor in the cascade of cell death events which may originate from the stroke brain and migrating to the heart. Although the exact mechanism linking cerebral ischemia with cardiac damage is not fully understood, further investigations are required to understand the overlapping molecular, cellular, and anatomical alterations seen in the heart following cerebral ischemia.

A clinically relevant model, such as the non-human primate (NHP) model of ischemia, may allow a better understanding of the cerebrovascular and cardiovascular event. Here, we demonstrated that brain and heart tissues harvested from NHPs that suffered transient global ischemia (TGI) months earlier displayed degenerative and inflammatory markers, further advancing the concept that distal secondary injuries and the development of chronic neuropathological manifestations propagate to both the brain and heart. In the clinic, such a brain-heart cell death crosstalk may indicate an integration of cerebrovascular and cardiovascular diagnostic assessments into standard clinical treatment and management of both diseases.

## RESULTS

### TGI decreases the volume of calbindin+ Purkinje cells in cerebellar lobules

**Figure 1:**
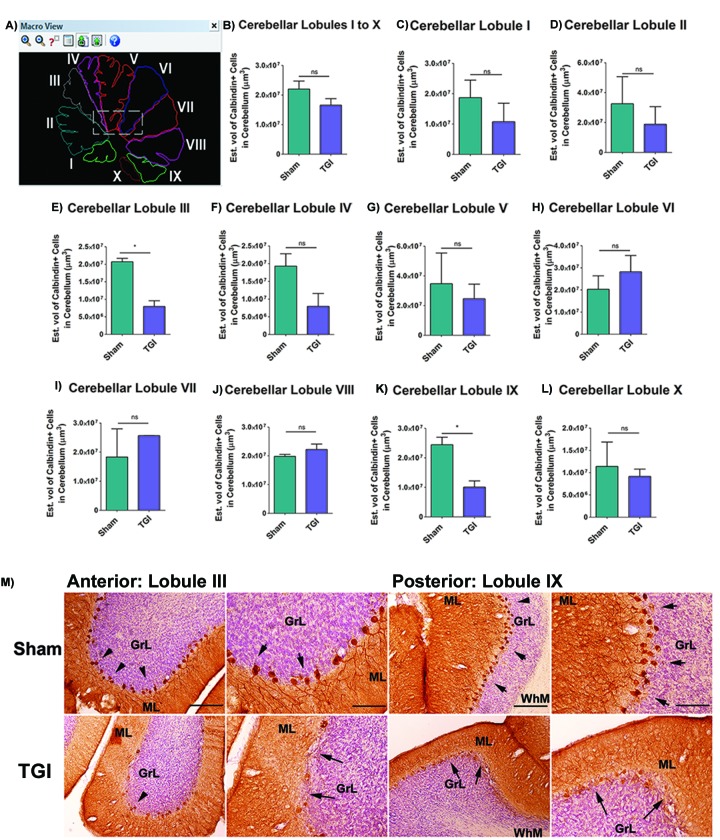
Increased Purkinje cell loss in cerebellar lobules after TGI **A.** Representative photomicrographs correspond to the outline of individual lobules of the cerebellum that were used for stereological analysis. **B.**-**L.** Quantitative analyses of the total number of Purkinje cells in each individual lobule of the cerebellum revealed significant cell loss in lobules III **E.** and IX (K) after TGI when compared to the respective lobules in the sham NHP (*p’s > 0.05). **M.** Representative photomicrographs of lobule III and lobule IX of TGI and sham animals taken from lateral sections of the cerebellum. Arrows denote positive staining for calbindin+ cells, which is characteristic of Purkinje cells residing in the cerebellum of TGI and sham animals. Scale bar: 50 µm. Student *t*-test, *p*’s > 0.05. Calbindin+ cells are expressed as estimated volume of positive cells. PCL: Purkinje cell layer; GCL: Granular cells layer; ML: Molecular layer. Data are expressed as mean ± SEM.

**Figure 2:**
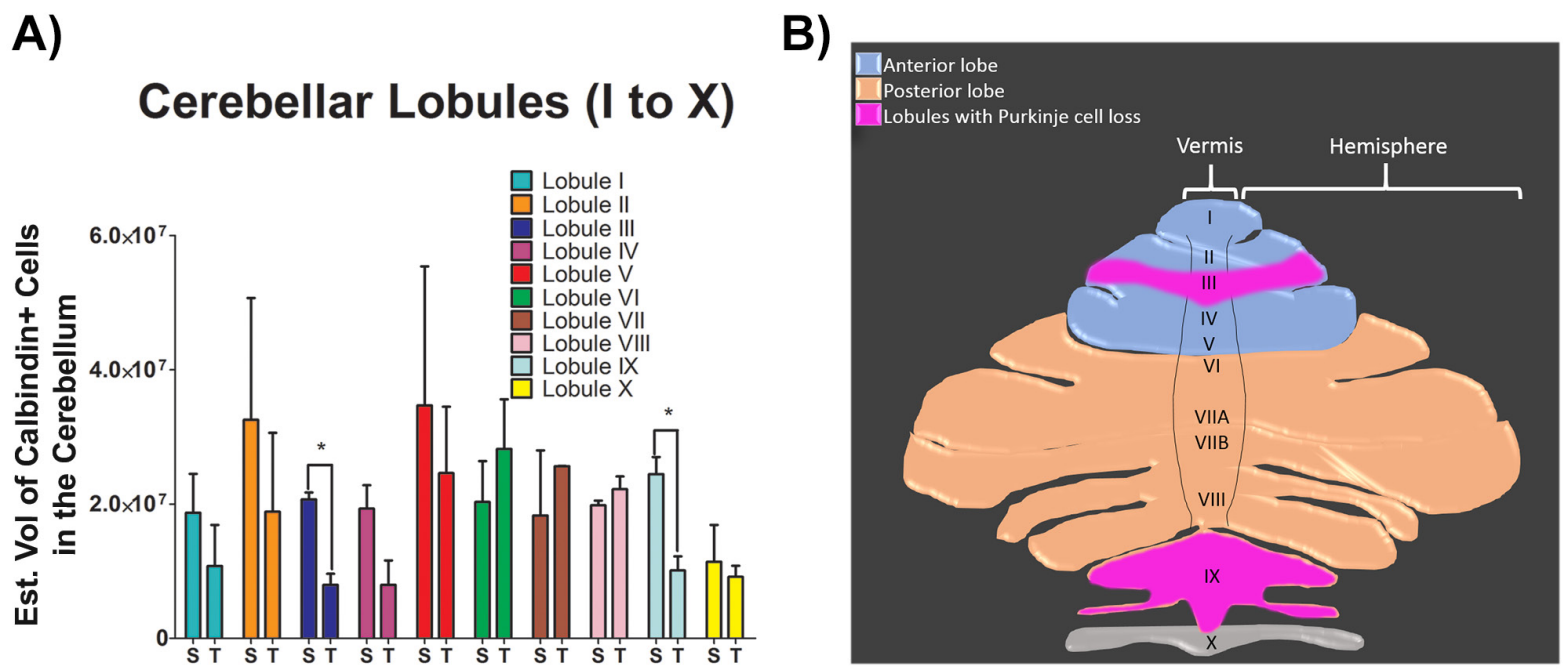
Total number of Purkinje cells in each individual lobule of the cerebellum after TGI **A.** Summary bar graph of quantitative analyses of total number of Purkinje cells in each individual lobule of the cerebellum. Analysis revealed significant cell loss in lobules III and IX after TGI when compared to the respective lobules in the sham NHPs (*p’s > 0.05). Student *t*-test, *p*’s > 0.05. Calbindin+ cells are expressed as estimated volume of positive cells. **B.** Schematic representation of the superior view of the cerebellum displaying the vermis, hemispheres and lobules. Pink highlighted areas represent the lobules with Purkinje cell loss post-TGI. Data are expressed as mean ± SEM.

Calbindin+ expression in every single lobule from the cerebellum was analyzed using immunohistochemistry and Cavalieri estimator techniques to reveal the secondary detrimental effects of delayed TGI on Purkinje cells. There were significant reductions in calbindin+ Purkinje cells in the cerebellum of the TGI NHPs, specifically in lobule III and lobule IX compared to the same areas in the cerebellum of the sham NHPs (Student *t*-test, *p*’s < 0.05), indicating TGI resulted in secondary damage to the cerebellum. In contrast, the expression of calbindin+ Purkinje cells in the other lobules of TGI NHP did not significantly differ compared to the respective lobules in the sham NHPs (Student *t*-test, *p*’s > 0.05) (Figure 1A-1M Figure 2), indicating that that calbindin cell loss was more pronounced in lobule III and lobule IX of the cerebellum at six months post-TGI.

### Increased TUNEL+ expression in the cerebellum after TGI positively correlates with Purkinje cell loss

**Figure 3:**
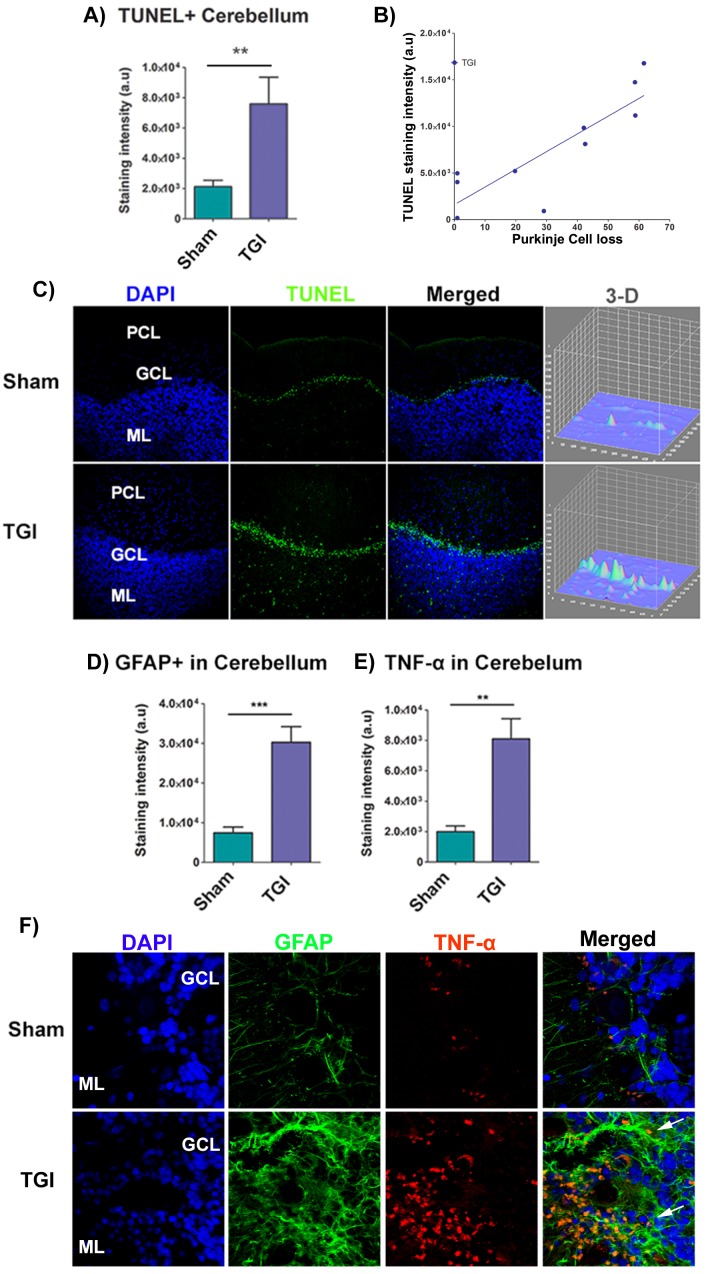
Increased apoptotic cells, astrogliosis, and TNF-α in the cerebellum after TGI **A.** Quantitative analysis of TUNEL expression in the granule layer of the cerebellum revealed a significant upregulation of TUNEL intensity after TGI relative to sham (*p < 0.05). **B.** Graph illustrates the correlation between TUNEL staining intensity and percent (%) of Purkinje cell loss in each lobule. There was a significant positive correlation between TUNEL staining intensity and percent of Purkinje cell loss in vulnerable lobules (*R*^2^ = 72.18; Pearson’s *r* = 0.8496). **C.** Representative confocal photomicrographs of positive expression of TUNEL (green) and Hoechst (blue) in the granule layer of the cerebellum of sham (top panel) and TGI (bottom panel), showing increased expression of TUNEL in the cerebellum of TGI relative to sham. Scale bar: 50 µm. Student *t*-test, *p* < 0.05. Data are expressed as mean ± SEM. **D.**, **E.** Fluorescent intensity analysis of GFAP+ cells and TNF-α in the cerebellum revealed significant upregulation of GFAP+ cells in the cerebellum of the TGI NHP compared to the sham NHP (*p*’s < 0.05). **F.** Representative photomicrographs correspond to lateral cerebellar sections showing the molecular and granule layers of the cerebellum stained with GFAP (astrocyte marker) and TNF-α (pro-inflammatory cytokine marker) after TGI. Arrows indicate positive staining for GFAP+ cells and TNF-α+ expression. Scale bar = 50 µm. Student *t*-test, *p*’s < 0.05. PCL: Purkinje cell layer; GCL: Granular cells layer; ML: Molecular layer. Data are expressed as mean ± SEM.

TUNEL expression in the cerebellum was analyzed using immunofluorescence to reveal the necrotic/apoptotic effect of delayed TGI NHPs. The mean TUNEL expression was markedly increased in all cerebellar lobules of the TGI NHPs compared to all cerebellar lobules from the sham NHPs (Student t-test, *p* < 0.05), suggesting a rampant cell death throughout the cerebellar region at this chronic phase post-ischemia. Pearson’s correlation analysis between TUNEL+ staining intensity of each individual cerebellar lobule and the percent of Purkinje cell loss of each individual cerebellar lobule revealed a positive significant correlation (*R*^2^ = 72.18; Pearson’s *r* = 0.8496) ( Figure 3A, 3B, 3C).

### Increased gliosis and pathological TNF-α expression in the cerebellum after TGI

GFAP and TNF-α expression in the cerebellum was analyzed using immunofluorescence techniques to reveal chronic secondary neuroinflammation in the cerebellum of the delayed TGI model in NHPs. The mean staining intensity of GFAP and TNF-α of the cerebellum revealed a significant pathological increase in the granule cell layer of the cerebellum of the delayed TGI NHPs relative to the cerebellum of the sham NHPs (Student *t*-test, *p*’s < 0.05) (Figure 3D, 3E, 3F). These results indicate persistent neuroinflammation in this chronic TGI NHP model.

### Increased monocyte HLA-DR+ and macrophage CD68+ cells expression in the cerebellum after TGI

**Figure 4:**
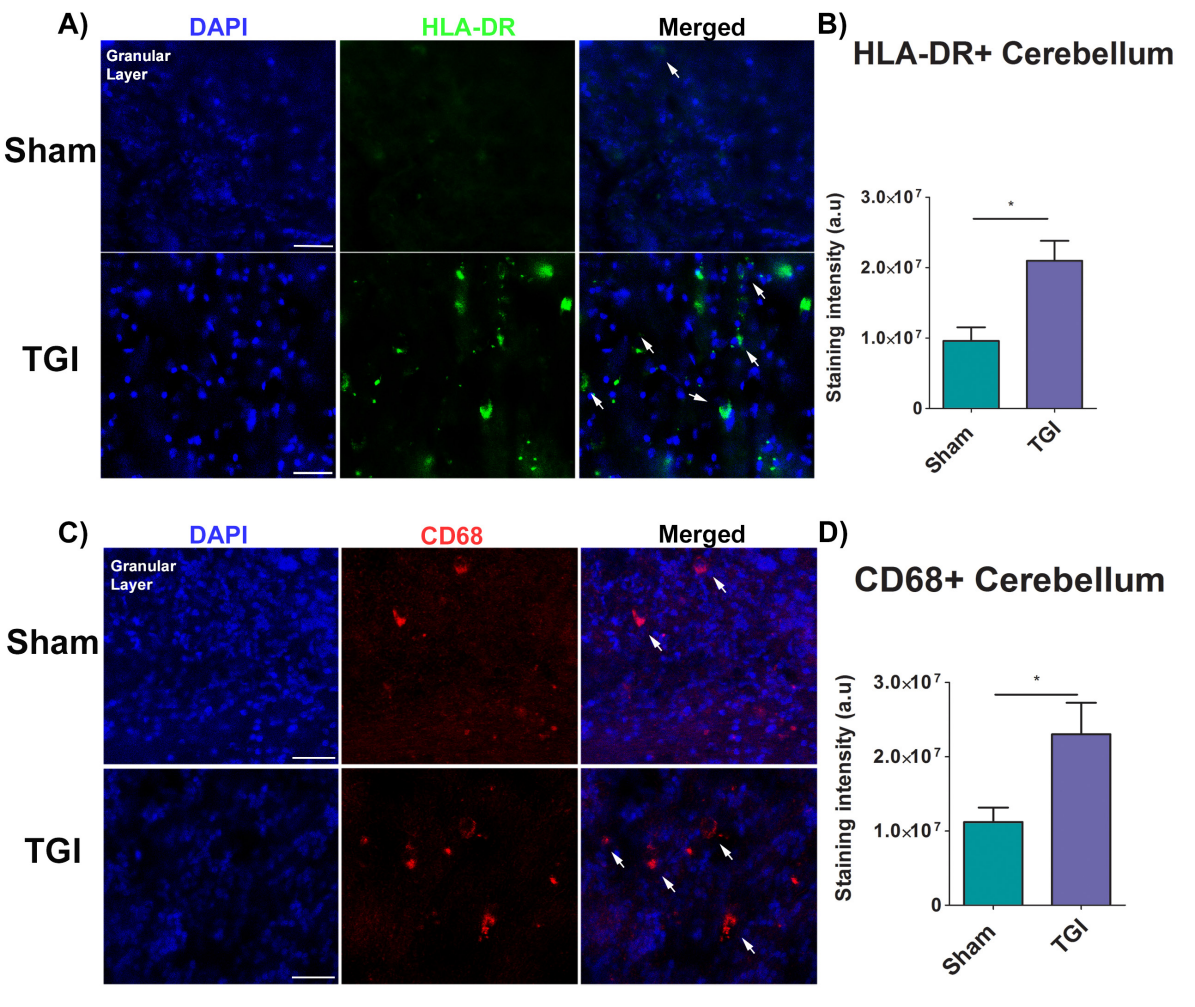
Increased expression of inflammatory markers in the granular cell layer of the cerebellum after TGI **A.**, **C.** Representative photomicrographs of the granular cell layer of the cerebellum. **B.** Fluorescent intensity examination revealed significant overexpression of HLA-DR cells in the granular cell layer of the cerebellum of the TGI NHP when compared to sham NHP (*p*’s < 0.0001). **D.** Fluorescent intensity examination revealed significant upregulation of macrophages (CD68+ cells) in the granular cell layer of the cerebellum of the TGI NHP when compared to sham NHP (*p*’s < 0.05). Scale bar = 50 µm. Student *t*-test, *p*’s < 0.0001. Data are expressed as mean ± SEM.

HLA-DR and CD68 expression in the cerebellum, specifically in the granular layer, was analyzed using immunofluorescence techniques to reveal markers of immune response and macrophages, respectively. The mean staining intensity in the cerebellum revealed a significant increased expression of HLA-DR and CD68+ macrophages in the granular layer of the delayed TGI NHPs relative to the granular layer of the sham NHPs (Student *t*-test, *p*’s < 0.0001) (Figure 4A, 4B, 4C). These results indicate an upregulation of the immune response in the cerebellum at several months after TGI.

### Increased monocyte HLA-DR+ and caspase 3+ expression in the heart after TGI

**Figure 5:**
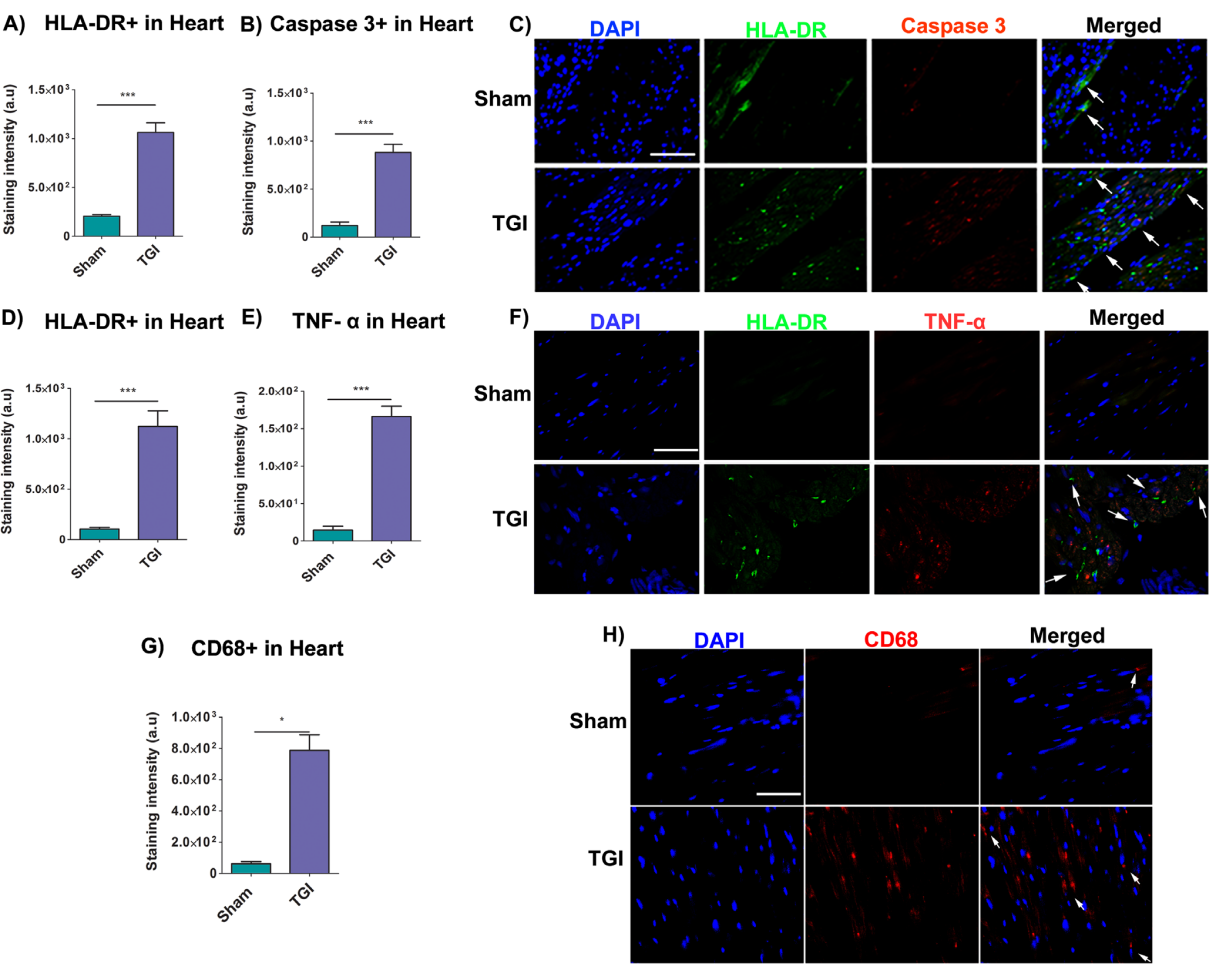
Increased expression of HLA-DR, caspase 3, TNF-α and CD68 in the heart after TGI Fluorescent intensity analysis revealed significant upregulation of HLA-DR cells **A.** and caspase 3 **B.** in the left ventricle of the TGI NHP heart when compared to the left ventricle of the sham NHP heart (*p*’s < 0.05). **C.** Representative photomicrographs of a left ventricle section of the heart showing positive expression of HLA-DR (human leukocyte marker) and caspase 3 (upstream regulator of apoptosis) after TGI. Scale bar = 50 µm. Student *t*-test, *p*’s < 0.0001. Data are expressed as mean ± SEM. Fluorescent intensity analysis demonstrated significant upregulation of HLA-DR **D.** and TNF-α **E.** in the left ventricle of the TGI NHP heart when compared to the left ventricle of the sham NHP heart (*p*’s < 0.05). **F.** Representative photomicrographs of a left ventricle section of the heart showing positive expression of HLA-DR (human leukocyte marker) and TNF-α (pro-inflammatory cytokine marker) after TGI. Similarly, fluorescent intensity quantifications demonstrated significant upregulation of macrophages (CD68+ cells) in the left ventricle of the TGI NHP heart relative to the left ventricle of the sham NHP heart **G.** (*p*’s < 0.05). **H.** Representative photomicrographs of a left ventricle section of the heart showing positive expression of CD68+ macrophages post TGI. Scale bar = 50 µm. Student *t*-test, *p*’s < 0.0001. Data are expressed as mean ± SEM.

HLA-DR and caspase 3 expression in the heart, specifically in the left ventricle was analyzed using immunofluorescence techniques to reveal markers of immune response and upstream regulator of apoptosis, respectively. The mean staining intensity in the heart revealed a significant increased expression of HLA-DR and caspase 3 staining in the left ventricle of the delayed TGI NHPs relative to the left ventricle of the sham NHPs (Student *t*-test, *p*’s < 0.0001) (Figure 5A, 5B, 5C). These results indicate a robust cardiac immune response and apoptotic response was detectable even at several months after TGI.

### Increased monocyte HLA-DR+ and TNF-α+ expression in the heart after TGI

To capture an interaction between immune response and inflammation in the heart as potential pathway of the observed apoptotic cardiac cell death, we next examined immunofluorescent expression of HLA-DR and TNF-α in the heart, specifically in the left ventricle. The mean staining intensity in the heart revealed a significant increase expression of HLA-DR and TNF-α in the left ventricle of the delayed TGI NHPs relative to the left ventricle of the sham NHPs (Student *t*-test, *p*’s < 0.0001) (Figure 5D, 5E, 5F).

### Increased CD68+ cells expression in the heart after TGI

Similarly, CD68 expression in the heart samples was analyzed using immunofluorescence techniques to reveal the presence of macrophages. The mean staining intensity in the heart revealed a significant increased expression of CD68+ macrophages in the delayed TGI NHPs relative to sham NHPs (Student *t*-test, *p*’s < 0.0001) (Figure 5G, 5H). These results implicate a robust cardiac immune response detectable even at several months after TGI.

## DISCUSSION

In the present study, we investigated the degenerative consequences of global cerebral ischemia model in distal areas including the cerebellum and the heart. Six months after TGI, detailed analyses of the NHP cerebellum indicated a massive reduction in Purkinje cell survival within cerebellar lobule III and lobule IX. Further analysis revealed widespread and significant apoptotic and inflammatory responses in the TGI NHP cerebellum. Similarly, we detected a significant increase in specific pro-cell death immune, apoptotic, and inflammatory markers in the heart in chronic phase following TGI. Altogether, these results suggest that the originally reported hippocampal cell death as the primary ischemic injury is not the only pathological consequence following TGI, but there are also secondary injury events in distal regions, such as the cerebellum and the heart that may persist over a prolonged period post-ischemia.

In the cerebellum, the expression of calbindin, which is specific marker for Purkinje cells, displayed a localized decrease in lobule III and IX in the cerebellum. TUNEL analysis confirmed the widespread apoptotic signal within the cerebellum relative to sham cerebellum. The brain may represent the most sensitive organ to hypoxia or ischemic injuries [23]. Several distinct characteristics of the brain may contribute to its increased susceptibility to low oxygen levels, including its high oxygen and glucose consumption coupled with low storage levels of glucose/glycogen and high metabolic functions [24, 25]. Interestingly, neural germinal zones like SVZ, the hippocampus, and even parts of the cerebellum at postnatal periods [26-31], are fenestrated with massive supply of vasculature and oxygen-supporting cells, such as pericytes, which may contribute to the stemness fate and functional phenotype of neural progenitor cells (NPCs). Delivery of oxygen to neurogenic niches is of critical importance for their proliferative, and inmunomodulatory function, in fact, HIF-1a is upregulated when low oxygen levels are detected, thus activating angiogenic and vasculogenic pathways to increase blood flow [32]. However, this endogenous response is not sufficient to arrest the cell death cascade. Accordingly, despite such complex vascular fenestrations of the neurogeneic niches, these unique oxygen-dependent features may indicate a higher demand for energy metabolism, increasing the vulnerability of these regions during ischemic conditions. In parallel, cultured endothelial cells secrete factors to stimulate proliferation of neural precursor and induced their differentiation to a neuronal fate [33], suggesting that targeting these neurogenic niches with exogenous stem cells may boost the endogenous neurogenesis thereby abrogating pathological conditions, such as ischemic insults. Previous studies have demonstrated the susceptible nature of cerebellar Purkinje cells following ischemic insult, which is partly due to their high metabolic activity [34-37]. Downregulated levels of neuronal metabolism by blood flow in cerebellar hemisphere are frequently reported on positron-emission tomography (PET) and single-photon emission CT (SPECT) in stroke patients [38]. In unilateral stroke, this phenomenon is referred to as crossed cerebellar diaschisis or CCD. After stroke, there is a marked depression of metabolic function and activity manifested as reduced blood flow in the cerebellar hemisphere contralateral to a supratentorial infarct which it is likely to interrupt the corticopontocerebellar fibers and to cause a downregulation of synaptic cerebellar Purkinje cell function and perfusion [38, 39]. Interestingly, long term chronic CCD instigate cerebellar volume loss in patients after supratentorial ischemic infarct [39]. Such phenomenon of diaschisis in patients has been documented in stroke animals [40, 41]. A topographic study has revealed anatomical and functional hallmarks of specific cerebellar lobules [42]. Lobule III, a key component in the anterior lobe of the cerebellum, is predominantly responsible for leg and foot sensorimotor function [42-44]. Meanwhile, lobule IX is located in the posterior cerebellar lobe and functions in the visual guidance of movement, thereby utilizing visual information to direct movements and adjust reflexes appropriately [42-46]. Lesions affecting the anterior lobe vermal lobules I-III as well as posterior lobules VIII and IX can result in balance and gait instability [42]. Clinically, balance and gait disability are a common secondary condition following cerebral ischemia [44-53], and could potentially be reflected in the present observation of distal neurodegeneration in the cerebellum seen in our TGI NHPs.

Furthermore, exacerbation of GFAP or astrogliosis along with secretion of pro-inflammatory cytokines, including TNF-α, are known to be highly expressed in the central nervous system during neurologic diseases associated with inflammation [54-57]. GFAP and TNF-α immunofluorescent staining showed increased expression in the area of the granule layer of the cerebellum, which further supports the ongoing secondary damage and inflammatory events during delayed ischemia. In line with our findings, previous studies have revealed that hypoxic-ischemic injured rats often present a significant increase in GFAP-positive cell density in posterior region of the brain and further distally to the cerebellar cortex [57]. Clinical studies have documented similar exacerbation of reactive astrocytosis in the brains of human infants following perinatal hypoxic-ischemic insult [58, 59]. The significant increase in TNF-α in the cerebellum is consistent with previous studies detailing a strong and persistent inflammatory response following ischemic insult [60-63].

Reactive astrocytosis and secretion of pro-inflammatory cytokines further reinforce the primary inflammatory response through a mechanism of delayed secondary damage in the cerebellum, which is suggestive of the vulnerable nature of the cerebellum as a target of delayed ischemic and hypoxic insult. Remarkably, cerebral ischemia and delayed cardiac failure are substantial causes of mortality and disability around the world [64-70]. We, and others, have implicated a pathologic link between the cerebral ischemia and cardiac cell death in rodents [7, 11, 65]. Here, we demonstrated that TGI in NHPs can similarly lead to secondary injury and harmful modification to cardiac cells. In the heart of TGI-exposed animals, the apoptotic marker caspase 3 and the inflammatory marker TNF-α were significantly upregulated compared to the sham heart. Of note, we observed a significant upregulation of these apoptotic and inflammatory markers specifically in the ventricles and throughout the intraventricular septum. Certain pathologic conditions can alter the pattern and function of myocardial contractility [66-79]; however, there is still much to be uncovered about the mechanism in which cerebral ischemia affects heart function. Currently, we recognize that a significant physiological change occurs *via* the inability of the ischemic brain to control heart rate, which is suggestive of an autonomic relationship between the ischemic brain and compromised heart function [12, 71, 80]. In addition, studies have revealed that insular cortex damage may disrupt this autonomic balance that can subsequently lead to potentially fatal arrhythmias and death [74-81]. Thus, the observed significant increments in caspase 3 and TNF-α are highly suggestive that an indirect pathological pathway of cell death exists between the ischemic brain and the heart in the chronic phase following TGI.

Although we detected here the presence of inflammatory markers within the cerebellum and the heart, these “inflamed cells” likely only correspond to a small number of inflammatory cells that manifest within the brain and periphery. However, because cell death is a dynamic process, even the minimum level of inflammatory response may represent a positive-feedback loop that can eventually excite the formation of new inflammatory and immune cells to create a variety of mediators including pro- cell death cytokines that are able to directly act on the brain and peripheral organs [82]. The notion that anesthesia may preclude ischemia-like symptoms in the brain and the heart has been recognized [83-85], thus a caveat of the study is that since the present animals were subjected to prolonged general anesthesia, then it is possible that some of the pathological symptoms seen here may be due to anesthesia effects. However, our study involved a chronic survival (i.e., 6 months post-TGI), and the control animals were also subjected to prolonged anesthesia, which should eliminate many of the reported anesthesia effects contributing to the observed pathological outcomes. We believe that further analysis of immunomodulatory cells of the NHP brain and peripheral organs will enhance our knowledge about the evolution of neurodegenerative secondary injuries at delayed time points in ischemia-plagued neurological diseases.

The current study supports a pathologic link between ischemia in the CNS and secondary cell injury in both distal brain regions (cerebellum) and peripheral organs (heart) which may further contribute to the delayed functional impairments in survivors of global ischemia. We previously reported this brain-heart interaction in experimental stroke model in rodents [7]. In this study, we were able to extend this pathologic relationship to a larger animal model by using NHPs. This translational approach further advances our understanding of the molecular, cellular, and anatomical modifications that occur following ischemia, and will likely open new avenues of research for developing novel therapies that encompass these overlapping pathological brain and heart manifestations inherent in cerebrovascular and cardiovascular diseases.

## MATERIALS AND METHODS

### Subjects

Brain and heart tissues used in the present histological and immunohistochemical studies were obtained from a separate cohort of animals that belonged to our original study [5] which underwent the same TGI procedure, but were allowed to survive up to 6 months post-surgery. Eight adult (about 8 years of age) Rhesus (Macaca mulatta), weighing about 4.8 to 6.0 kg were used on the present study. All experimental procedures were approved by the Institutional Animal Care and Use Committee in accordance with the NIH Guide for the Care and Use of Laboratory Animals (Bethesda, MD, USA). The animals were housed in an Association for Assessment and Accreditation of Laboratory Animal Care International-accredited NHP facility (57-R-0002). NHPs were housed in cages in a temperature- and humidity-controlled room that was maintained on 12/12 hour-light/dark cycles. They had free access to food and water. All necessary steps were performed to minimize animal pain and stress throughout the study. All the immunocytochemical and quantification analysis were performed double blinded. Experimental and control group information was withheld from the investigators throughout the study. Eight NHPs (Macaca fuscata) were either exposed to sham (*n* = 4) or transient global ischemia (TGI; *n* = 4) [5].

### Stroke surgery

Stroke surgery was performed using the transient global ischemia (TGI) technique as described in previous studies [86-88]. NHPs were anesthetized with a mixture of 1-2% halothane mixed with 40% O2 and 60% N2O *via* a face mask, and body temperature was maintained at 37± 0.3°C during the surgical procedures. Using thoraco-laparotomy technique, the brachiocephalic and left subclavian arteries were exposed, and subsequently clipped for 20 min just distal to the bifurcation from the aortic arch. For sham NHP surgery, the procedure involved anesthetizing the animal and exposing and isolating the right brachiocephalic and left subclavian arteries without clipping or inserting the filament. Thereafter, incisions were closed and animals were allowed to recover from anesthesia. Please see table for neurological and histological observations in Supplementary Materials. All animals were euthanized at 6 months post-surgery for subsequent analysis.

### Animal status and physiological parameters

The baseline for physiological parameters (before clamping the arteries) was as follows: mean arterial blood pressure, 86 ± 12 (mean SD) mm, Hg: PaO2, 174 ± 29 mm Hg: PaCO2, 32 ± 2 mm Hg: HCO3, 30 ± 6 mm Hg: SaO2, 99 ± 0.2%: pH, 7.5 ± 0.1: rectal temperature, 37 ± 0.5°C: and blood glucose concentrations, 90 ± 20 mg/dl. Immediately after clamping the arteries, all NHP displayed pupil dilation and the mean arterial blood pressure increased to 170 ± 10 mm Hg. However, immediately after reperfusion, NHP became normotensive meaning that all the physiological parameters became normal similar to baseline levels. Before clamping of the arteries, the cerebral blood flow was constant at 40 ml/100 g brain/min and during ischemia (clamping of the arteries) it decreased to 0-1 ml/100 g brain/min. NHP showed uneventful recovery within 1-3 h post-ischemic insult.

### Brain and organ harvesting, fixation, and sectioning

Under deep anesthesia, NHPs were re-anaesthetized and perfused transcardially. Briefly, NHPs were perfused through the ascending aorta with 500 mL of saline, followed by 1000 mL of 4% paraformaldehyde in phosphate buffer (PB). The cerebellum and the hearts were removed and post-fixed in the same fixative for 24 h, followed by 30% sucrose in PB until completely sunk. Series of lateral sections were collected throughout the cerebellum (the vermis and the hemisphere) at a thickness of 40µm using with a cryostat and then stored at -20°C in cryoprotectant solution. Series of transverse sections from the short axis of the base (ventricles) to the apex at a thickness of 40µm were collected and mounted directly onto slides and storage at -20°C.

### Immunohistochemistry

Cerebellar Purkinje neurons were labeled using anti-calbindin antibody. Staining was conducted on every 1/6 lateral section of the vermis and cerebellar hemispheres. In all NHPs, sections were anatomically matched. Series of 6 sections per NHP were processed for calbindin staining. Six free-floating coronal sections (40 µm) were washed 3 times in 0.1M phosphate-buffered saline (PBS) to clean the section from the cryoprotectant. Afterwards, all sections were incubated in 2% hydrogen peroxide (H2O2) and 40% methanol solution for 20 minutes and washed 3 times with 0.1M PBS for 10 minutes each wash. Next, all cerebellar sections were incubated in blocking solution for 1 hour using 0.1M PBS supplemented with 10% normal goat serum and 0.1% Triton X-100. Sections were then incubated overnight at 4°C with rabbit anti-calbindin (1:300; Cell signaling, 2173), antibody markers in 0.1M PBS supplemented with 3% normal serum and 0.1% triton X-100. Sections were then washed 3 times with 0.1M PBS and incubated in biotinylated goat anti-rabbit secondary antibody (1:200; Vector Laboratories, Burlingame, CA) in 0.1M PBS supplemented with normal goat serum, and 0.1% Triton X-100 for 1 hour. Next, the sections were incubated for 60 minutes in avidin-biotin substrate (ABC kit, Vector Laboratories, Burlingame, CA) and washed 3 times with 0.1M PBS for 10 minute each wash. All sections were then incubated for 1 minute in 3, 30-diaminobenzidine (DAB) without metal enhancer (Vector Laboratories) and washed 3 times with 0.1M PBS for 10 minutes each wash. Sections were then mounted onto glass slides, dehydrated in ascending ethanol concentration (70%, 95%, and 100%) for 2 minutes each and 2 minutes in xylenes, and cover-slipped using toluene as mounting medium.

### Immunofluorescent staining

Staining for GFAP and TNF-α positive cells was conducted on every 1/6 sections, 40 µm thick, lateral sections from the vermis and cerebellar hemispheres. Cerebellar sections were washed three times for 10 minutes in 0.1M PBS. Six sections were incubated with saline sodium citrate (SSC) solution at PH 6 for 40 minutes at 80° C for antigen retrieval. Then, samples were blocked for 60 min at room temperature with 8% normal goat serum (Invitrogen, CA) in 0.1M PBS containing 0.1% Tween 20 (PBST) (Sigma). Sections were then incubated overnight at 4°C with rabbit polyclonal anti-human TNF-α (1:200; life technologies, PA1-26810) and chicken polyclonal anti-human GFAP (1:100; Abcam, ab4674) with 3% normal goat serum. Then, the sections were washed five times for ten minutes in 0.1M PBST and soaked in 5% normal goat serum in 0.1M PBST containing corresponding secondary antibodies, goat anti-rabbit IgG-Alexa 594 (red) (1:1500; Invitrogen), and in goat anti-chicken IgG-Alexa 488 (green) (1:400; Invitrogen) for 90 minutes. Finally, cerebellar sections were washed five times for ten minutes in 0.1M PBST and three times for five minutes in 0.1M PBS, processed for 1:300 Hoechst 33258 (bisBenzimideH 33258 trihydrochloride, Sigma) for 30 min, washed in 0.1M PBS, and cover-slipped with Fluoromount (Aqueous Mounting Medium; sigma F4680). Cerebellar sections were examined using a confocal microscope (Olympus). Control studies included exclusion of primary antibody substituted with 5% normal goat serum in 0.1M PBS. No immunoreactivity was observed in these controls.

HLA-DR alpha is one of the HLA class II alpha chain paralogues. It plays a central role in the immune system by presenting peptides derived from extracellular proteins. Adaptive immunity is mediated by T cells, and its effectors CD8+T cells, B cells and activated macrophages (M1) expressing HLA or MHC II antigen presenting cells and IFN-γ altogether instigate an inflammatory response to injury. The present immunostaining focused on characterizing this Th1/M1 pro-inflammatory response using HLA-DR+ cells as markers of inflammation. Staining for inflammation was conducting by targeting ED1/CD68 and HLA-DRA and TNF-α on every 1/6 of the vermis, cerebellar hemispheres and transverse sections from the base of the heart to the apex. In all animals, sections were anatomically matched. Series of 6 to 4 sections per NHP were processed for ED1/CD68 and HLA-DR staining. Four transverse sections of the base of the heart whereby the ventricles are easily noted (40 µm) were washed 6 times in 0.1M phosphate-buffered saline (PBS) to clean the section from the cryoprotectant. Afterwards, sections were incubated with saline sodium citrate (SSC) solution at PH 6 for 40 minutes at 80°C for antigen retrieval. Later, sections were subjected to 2% hydrogen peroxide (H2O2) solution for 20 minutes and washed 3 times with 0.1M PBS for 10 minutes each wash. Next, all sections were incubated in blocking solution for 1 hour using 0.1M PBS supplemented with 5% normal goat serum (Invitrogen, CA) and 0.1% Triton X-100. Sections were then incubated overnight at 4°C with mouse anti-human CD68 (1:100 Bio-rad MCA341), HLA-DR (1:600 Dako M0746), and rabbit polyclonal anti-human TNF-α (1:200; life technologies, PA1-26810antibody markers in 0.1M PBS supplemented with 3% normal serum and 0.1% triton X-100. Sections were then washed 3 times with 0.1M PBS and incubated in in goat anti-mouse IgG-Alexa 488 (green) (1:400; Invitrogen) and goat anti-rabbit igG-Alexa 594 (red) (1:1500; Invitrogen) in 0.1M PBS supplemented with normal goat serum, and 0.1% Triton X-100 for 1 hour and washed 3 times with 0.1M PBS for 10 minute each wash. Finally, sections were washed five times for ten minutes in PBST and three times for five minutes in PBS, processed for Hoechst 33258 (bisBenzimideH 33258 trihydrochloride, Sigma) for 30 min, washed in PBS, and cover-slipped with Fluoromount (Aqueous Mounting Medium; sigma F4680).

Four transverse sections of the base of the heart whereby the ventricles are easily noted (40 µm) were washed 6 times in 0.1M phosphate-buffered saline (PBS) to clean the section from the cryoprotectant. Afterwards, sections were incubated with saline sodium citrate (SSC) solution at PH 6 for 40 minutes at 80°C for antigen retrieval. Later, sections were subjected to 2% hydrogen peroxide (H2O2) solution for 20 minutes and washed 3 times with 0.1M PBS for 10 minutes each wash. Next, all sections were incubated in blocking solution for 1 hour using 0.1M PBS supplemented with 5% normal goat serum (Invitrogen, CA) and 0.1% Triton X-100. Sections were then incubated overnight at 4°C with mouse anti-human HLA-DR (human leukocyte antigen; 1:600 Dako M0746), and rabbit polyclonal anti-human caspase 3 (1:250) antibody markers in 0.1M PBS supplemented with 3% normal serum and 0.1% triton X-100. Sections were then washed 3 times with 0.1M PBS and incubated in goat anti-mouse IgG-Alexa 488 (green) (1:400; Invitrogen) and goat anti-rabbit igG-Alexa 594 (red) (1:1500; Invitrogen) in 0.1M PBS supplemented with normal goat serum, and 0.1% Triton X-100 for 1 hour and washed 3 times with 0.1M PBS for 10 minute each wash. Finally, sections were washed five times for ten minutes in PBST and three times for five minutes in PBS, processed for Hoechst 33258 (bisBenzimideH 33258 trihydrochloride, Sigma) for 30 min, washed in PBS, and cover-slipped with Fluoromount (Aqueous Mounting Medium; sigma F4680).

### Stereological analysis: Cavalieri estimator

Unbiased stereology was performed on cerebellar sections immunostained with calbindin to estimate the Purkinje cells volume. Sets of 1/6 section, ∼6 systematically random sections, of about 240 µm apart, were taken from the cerebellum in TGI and sham cerebellum. Of note, section thicknesses were confirmed as being between 20.0-21.0 microns after dehydration, and this did not statistically differ between groups. Calbindin positive cells were examined using the Cavalieri estimator probe of the unbiased stereological cell technique 19 revealing the volume of calbindin positive neurons in the cerebellum. All samplings were optimized to count at least 300 cells per cerebellar hemisphere with error coefficients less than 0.07. The Cavalieri estimator was executed using a point grid spaced equally both across and down directions. A grid space of 100 µm was used in order to cover the different gray and white matter regions each one representing our region of interest (ROI) [88].

### Analysis of fluorescent staining

From all sections, approximately 4 to 6 images of 20X magnification were taken from each lateral cerebellar section and from each transverse sections of the heart using confocal microscopy (Olympus) and analyzed with ImageJ (National Institutes of Health, Bethesda, MD). All photomicrographs were converted to gray scale. Background was selected from blank control images, and subsequently used to subtract the background from all images. The same threshold was used for all images. Thereafter, the staining intensity of each section was quantified as the average optical density readings of 4 randomly selected areas within that section. The final staining intensity of each group resulted as the average of each staining intensity per section.

### Statistical analysis

All data were expressed as mean ± SEM and statistically evaluated using two-way ANOVA followed by Bonferonni’s test (GraphPad version 5.01). In addition, Student’s *t*-tests were also used to determine and compare the effect of TGI stroke NHP *versus* sham NHP. We used the Pearson correlation test to compare the alterations of volume densities of Purkinje Cells and TUNEL+ staining intensity. All comparisons were considered significant at *p* < 0.05.

## SUPPLEMENTARY MATERIALS



## Abbreviations

BNP: Brain natriuretic peptide
CRP: C-reactive protein
GFAP: Glial fibrillary acidic protein
HLA-DR: Human Leukocyte Antigen - antigen D Related
IgG: Immunoglobulin G
OGD: Oxygen glucose deprivation
NPCs: Neural progenitor cells
PBST: Phosphate buffer saline TX-100
PBS: Phosphate buffer saline
SSC: Saline sodium citrate
TNF-α: Tumor necrosis factor alpha
TUNEL: Terminal deoxynucleotidyl transferase dUTP nick end labeling
