# Supplementary material for: Chronic inflammation and apoptosis propagate in ischemic cerebellum and heart of non-human primates
Source: Oncotarget. 2017 Jun 1;8(61):102820–34. doi: 10.18632/oncotarget.18330 (PMC5732692; doi:10.18632/oncotarget.18330)
Supplement: Supplementary file 1 [file oncotarget-08-102820-s001.pdf]

Chronic inflammation and apoptosis propagate in ischemic cerebellum and heart of non-human primates

SUPPLEMENTARY MATERIALS

Neurological observations	Sham control	TGI
Partial paralysis and rigidity	No	Yes
Intermittent episodes of convulsions	No	Yes
Partial absence of motion on both arms and hindlimbs	No	Yes
Partial absence of reflex on upper extremities	No	Yes
Difficulty to right itself even after assistance	No	Yes
Mild pneumothorax	No	Yes
Partial inability to use digits and absence of grasping	No	Yes
**Histological Observations**	**Sham control**	**TGI**
CA1 Cell loss*	No	Yes
Increase TUNEL+ cells in hippocampus*	No	Yes
Elevated eosinophilic cell bodies in CA1 region*	No	Yes
GFAP+ cell infiltration in hippocampus*	No	Yes
Purkinje cell loss#	No	Yes
Increase of GFAP+ cells in cerebellum#	No	Yes
Upregulation of CD68+ (Macrophage) in cerebellum and heart#	No	Yes
Upregulation of HLA-DR+ cells in cerebellum and heart#	No	Yes
Overexpression of TNF-α in cerebellum and heart#	No	Yes
Overexpression of caspace 3+ cells in heart#	No	Yes
Upregulation of TUNEL + cells in cerebellum#	No	Yes

Legend: *Hara et al., 2007; #Present study
